# What factors explain the number of physical therapy treatment sessions in patients referred with low back pain; a multilevel analysis

**DOI:** 10.1186/1472-6963-5-74

**Published:** 2005-11-24

**Authors:** Ilse CS Swinkels, Raymond H Wimmers, Peter P Groenewegen, Wil JH van den Bosch, Joost Dekker, Cornelia HM van den Ende

**Affiliations:** 1 NIVEL Netherlands Institute for Health Services Research; PO Box 1568, 3500 BN Utrecht, the Netherlands; 2 Department of General Practice, Radboud University Medical Centre Nijmegen; Nijmegen, the Netherlands; 3 Department of Rehabilitation Medicine, Institute for Research in Extramural Medicine, VU University Medical Centre Amsterdam, Amsterdam, the Netherlands

## Abstract

**Background:**

It is well-known that the number of physical therapy treatment sessions varies over treatment episodes. Information is lacking, however, on the source and explanation of the variation. The purposes of the current study are: 1) to determine how the variance in the number of physical therapy treatment sessions in patients with non-specific low back pain (LBP) in the Netherlands is distributed over patient level, therapist level and practice level; and 2) to determine the factors that explain the variance.

**Methods:**

Data were used from a national registration network on physical therapy. Our database contained information on 1,733 patients referred with LBP, treated by 97 therapists working in 41 practices. The variation in the number of treatment sessions was investigated by means of multilevel regression analyses.

**Results:**

Eighty-eight per cent of the variation in the number of treatment sessions for patients with LBP is located at patient level and seven per cent is located at practice level. It was possible to explain thirteen per cent of all variance. The duration of the complaint, prior therapy, and the patients' age and gender in particular are related to the number of physical therapy treatment sessions.

**Conclusion:**

Our results suggest that the number of physical therapy treatment sessions in patients with LBP mainly depends on patient characteristics. More variation needs to be explained, however, to improve the transparency of care. Future research should examine the contribution of psychosocial factors, baseline disability, and the ability to learn motor behavior as possible factors in the variation in treatment sessions.

## Background

It is well-known that the number of physical therapy treatment sessions varies over treatment episodes [1-10] and it is important for health care policy makers, physical therapists and patients to gain greater understanding of the sources of this variation. Greater understanding will increase the transparency of care and can provide novel insights into the quality of care. On grounds of equity, an 'ideal' situation is one where health status is the main determinant of treatment choice and hence of variation. As a consequence, the variation is appropriate when it occurs due to 'need' factors like the patient's clinical health status [11], but it is questionable whether the variation is appropriate when it occurs due to factors like social structure, health beliefs, or enabling resources (such as accessibility). Elimination of inappropriate variation is necessary for quality improvement in physical therapy practice [12] and it is important to know exactly where variance is located if proper quality measures are to be implemented. The variance may be on different levels, including patient level, therapist level and practice level.

Few investigations have been made as yet into the reasons for the variation in the number of treatment sessions [5,13,14] and none of these distinguished between variation at patient level, variation at therapist level and variation at practice level. Hendriks et al. (2000) showed that the therapist's age, a specialization in manual therapy, and practice size were associated with fewer treatment sessions [5], but it remained unclear to what extent the amount of variation was explained and how it was distributed over the different levels. Other studies showed that the patient's age [5,14], the duration of the complaints [14], the therapist's diagnostic findings, the medical diagnosis [14], and additional claims for other health care services [13] were positively related to the number of treatment sessions. Information on the amount of variation at different levels is lacking in the above-mentioned studies and much of the variation remains unexplained, which means that these studies do not fulfill the need for clarification of variation in utilization of physical therapy. To the knowledge of the authors, the current study is the first one in which different levels are taken into account to estimate not only the variation, but also its location.

The aims of the current study are as follows: 1) to determine how the variance in the number of physical therapy treatment sessions in patients with non-specific low back pain (LBP) is distributed over patient level, therapist level and practice level; and 2) to determine the factors that explain the variation, with factors relating to all three levels being taken into account. We addressed our research questions to patients with LBP, since they form the largest population in physical therapy practices.

## Methods

### Registration network: sampling

Data from the National Information Service for Allied Health Care (called LiPZ in Dutch) are used for the current study. The National Information Service for Allied Health Care is a registration network of Dutch physical therapists working in private practices all over the country, and this network has been collecting health care-related data on a continuous basis since 2001. Physical therapists were invited to participate in the registration network in early 2001, the selection of therapists being based on practice-size and region. The therapists invited to take part were a sample of all private physical therapy practices as listed in a national database [15]. Our objective was a registration network of 40 practices and therapists could only participate if one of two specific software programs was used in their practice. Physical therapists with a homogeneous patient population (> 50% of the treatment episodes consisting of one patient category, such as children) were excluded from the network. Twenty per cent of the therapists invited to participate were eligible to take part. Frequently mentioned reasons for not participating were 'not enough time' and 'personal reasons'. When dropouts occurred, an a-selective procedure was used to invite new physical therapists to participate. A response rate of 20% is acceptable, considering the kind of research, since a long-term commitment and a computerized practice are factors that lower the response rate. Despite the relatively low response rate, comparisons with other available data show that the participating practices, therapists, and data collected appear to be representative for the Netherlands [16-18]. Over 140 physical therapists working in more than 60 practices have participated since 2001. Participants are offered some financial remuneration and they also receive benchmark data on an annual basis. Relevant information on the Dutch health care system is provided in Table 1.

**Table 1:** The organization of physical therapy in The Netherlands

In the Dutch health care system, physical therapists are only accessible after referral by a physician and over 90% of patients attending a physical therapist have been directly referred by their GP. The remaining 10% are referred by a medical specialist. People in the Netherlands have either public or private health insurance, depending on their level of income. Public insurance cover for physical therapy is nationally regulated and in 2002 and 2003 this meant that people with public insurance (66% of the population) and low back pain were covered for 9 treatment sessions per episode per year. People with public insurance were able to obtain additional private insurance that covered them for more than 9 treatment sessions. Private insurance cover (the other third of the population) for physical therapy was not regulated at national level. Every physical therapy session lasts about 25 minutes and physical therapists are paid per session, irrespective of the type of diagnosis and intervention. In the Netherlands, nearly all therapists working in primary care are organised in private practices. The Dutch situation will change in 2006; the differentiation between public and private health care insurances will disappear and physical therapy will be accessible without a referral.

### Registration network: methods

Dutch therapists in private practice generally use a software program to record their patients and treatments, and for reimbursement. In addition to the information regularly recorded, therapists participating in the network use special software to record supplementary information on all their patients. The selection of the data was based on the Dutch physical therapy guideline for clinical reporting, a guideline that specifies the data that are relevant for physical therapy practices. Participants submit their data on a monthly basis and the data are entered in the database after standardized quality control has been performed to check for missing or inconsistent data. The practice receives feedback in the case of missing or inconsistent data, and corrected data are entered in the database in the next month.

A written questionnaire, completed annually by all participants, provides information on characteristics of the participating therapists and practices and also includes questions about the attitude towards quality improvement. The feasibility of the Dutch LBP guideline [19] is specifically addressed. The question "Could you please rate your opinion of the feasibility of the Low Back Pain guideline? The rating can range from 0 (very bad) to 10 (excellent)" was used as indicator of the attitude of physical therapists towards the physical therapy guideline for Low Back Pain. All relevant variables collected are listed in Table 2.

**Table 2:** Overview of data collection

**Variables**	**Measurement**	**Used in analyses as**
***Demographic***		
Gender	Male; Female	Categorical
Age	Date of birth	Continuous: years old at start treatment episode
Health insurance	Public health insurance (Puhi), private health insurance (Prhi)	Categorical: Puhi; Prhi; Unknown
Education	Highest level of education: Primary school, secondary education, higher education, university	Categorical: Low (primary); Middle (secondary, higher); High (university); Unknown
Urbanization rate	1 very high, 2 high, 3 moderate, 4 low, 5 very low	Categorical: High (3+2+1); Low (5+4); Missing values (1.3%) recoded as high urbanization rate
		
*Complaints*		
Specialization of referring physician	GP or Medical specialist	Categorical: GP; Medical specialist
Reason for referral	As given by letter by the referrer; coded with ICPC (26) by researchers	Selection of patients with ICPC-code L03.00
Duration of complaint at start episode	< 2 days; 2–7 days; 1 week – 1 month; 1–3 months; 3–6 months; 6 months – 1 year; 1–2 years; > 2 years; unknown	Categorical: < 1 month; > 1 month; Missing values (1.0%) recoded as < 1 month
Recurrent complaint (appearing after a complaint-free episode of at least four weeks and at most two years)	Yes; No; Unknown	Categorical: Yes; No; Missing values (3.7%) recoded as no
Previous physical therapy for the same or other complaints in the last two years	Yes; No; Unknown	Categorical: Yes; No; Missing values (6.1%) recoded as no
		
*Treatment*		
Treatment goals	Based on the International Classification of Functioning, Disability and Health (27); One main goal (out of 24) at the level of physical functions; One main goal at the level of activities (out of 11)	5 dichotomous variables; Missing values (0.6%) recoded as changing body position
Interventions	Based on Dutch classification; three interventions at most (out of 25) applied in at least 50% of the sessions	5 dichotomous variables; Missing values (16.1%) integrated in reference category
		
*Therapists*		
Gender	Male; Female	Categorical: Male; Female; Missing values (2.1%) recoded as male
Age	Date of birth	Categorical: <45 years at January 1st, 2003; > 45 years at January 1st, 2003; Missing values (5.1%) recodes as > 45 years
Hours working per week	Patient-related number of hours	Categorical: <20 hours; 20–40 hours; >40 hours; Missing values (4.1%) recoded as 20–40 hours
Registration in quality register for manual therapy	Yes; No	Categorical: Yes; No
Additional training in LBP	Yes; No	Categorical: Yes; No; Missing values (4.1%) recoded as yes
Additional training in LBP guideline	Yes; No	Categorical: Yes; No; Missing values (4.1%) recoded as no
Feasibility of LBP guideline	1 item on questionnaire 10-point scale (1 = very bad; 10 = excellent; 7 = satisfactory)	Categorical: <7 points; >7 points; Unknown
Time since graduation	Date of graduation	Categorical: < 20 years since graduation; > 20 years since graduation; Missing values (6.2% recodes as > 20 years.
		
*Practices*		
Group size	Number of therapists	Categorical: Single handed; Group practice

### Study sample

Data from therapists who treated patients referred with non-specific LBP during the period July 2002-September 2003 were selected from the database for the current study; these data were supplied by 97 therapists in 41 practices. The therapists treated an average of 1.6 new patients with LBP per month (average in a 30-hour week). Twenty-four per cent of the 41 participating practices were solo practices (Table 3). The majority of the physical therapists were male; the mean age of the therapists was 43.5 years (sd 9.3). The therapists selected did not differ significantly from all Dutch physical therapists.

**Table 3:** Characteristics of therapists (n = 97) and practices (n = 41) in the sample and in the Netherlands (12,695 therapists)

		Sample				Dutch population of physical therapists [15]		
		%	(N)	Mean	(SD)	%	(N)	P

*Physical therapist*	Male	57.7	(56)			50.1	(6,359)	0.14
	< 45 years	50.5	(49)			56.4	(7,049)	0.32
	Registration quality register – manual therapy	12.4	(12)					
	Number of patient-related hours per week							
	< 20 hours	23.7	(23)					
	20–40 hours	61.9	(60)					
	> 40 hours	14.4	(14)					
	Additional training LPB	58.8	(57)					
	Additional training LBP guideline	39.2	(38)					
	Feasibility of the LBP guideline							
	< 7 points	39.2	(38)					
	> 7 points	43.3	(42)					
	Unknown	17.5	(17)					
	Number of new patients with LBP per therapist per month	1.6	(1.1)					
								
*Practice*	Single-handed	24.4	(10)					

Where the patient population is concerned, all patients aged 18 years or older who had been referred with LBP without radiation (ICPC-code L03.00) between July 2002 and September 2003 were selected from the database (n = 1,760). Patient data were collected until April 2004, at which time 1,733 of these 1,760 patients had a completed treatment episode (98.5%). Data relating to these 1,733 patients were used in the current study.

Ethical approval was not required, since patients only received the customary care and there were no experimental interventions for the purposes of the present study. Patients were nevertheless informed about the research project by posters and leaflets in the waiting rooms in the practices and patients could refuse to participate.

### Outcome variable and predictor variables

The outcome variable was the total number of treatment sessions per treatment episode. This variable was used as a continuous variable.

The predictor variables are listed in Tables 3 and 4. Age, gender, education level, health care insurance and urbanization rate are included as demographic variables. Variables relating to the complaints are also included, viz. the duration of the complaints, recurrent complaints, prior physical therapy or exercise therapy, and specialization of the referring physician. An interaction term consisting of gender and duration of the complaints was also added, since the gender distribution in patients with acute complaints was not equal to that in patients with chronic complaints. Treatment variables included variables on the treatment goals and the interventions. At the start of a treatment episode, therapists indicated one main treatment goal from a list of 11 predefined goals at activities level and/or one main treatment goal from a list of 24 predefined goals at physical functions level. Five treatment goals that were indicated in more than ten percent of the patients are included in the analyses as dichotomous variables. At the end of the treatment episode, physical therapists recorded a maximum of three interventions (from a list of 25 predefined interventions) that were applied in at least 50% of the treatment sessions. Interventions recorded in more than ten percent of the patients are included in the analysis as dichotomous variables (n = 5). Variables relating to gender, age, working hours per week, additional training in LBP and additional training in guideline-use for patients with LBP, the feasibility of the guideline LBP, registration in the quality register for manual therapy and group size were included at therapist and practice levels. Table 4 provides an overview of the characteristics of the variables at patient level.

**Table 4:** Characteristics of patients (n = 1,733)

		%	(No)	Mean	(SD)
**Demographic**					
Age in years				48.7	(16.3)
Male		45.2	(783)		
Education	Low	30.8	(534)		
	Middle	26.3	(456)		
	High	13.5	(234)		
	Unknown	29.4	(509)		
Health insurance	Public	56.7	(983)		
	Private	24.2	(420)		
	Unknown	19.0	(330)		
High urbanization rate (> 1,000 addresses per km^2^)^1^		58.9	(1,021)		
					
**Complaints**					
Duration complaint	< 1 month (acute)	48.2	(835)		
	> 1 month (chronic)	51.8	(898)		
Recurrent complaint^2^		47.0	(815)		
Previous physical therapy^3^		47.1	(817)		
Referred by general practitioner		95.2	(1,650)		
					
**Treatment**					
Treatment goal	Maintaining body position (yes)	18.1	(313)		
	Changing body position (yes)	19.0	(329)		
	Functions of mobility (yes)	39.6	(687)		
	Functions of muscles (yes)	14.3	(247)		
	Pain (yes)	11.4	(197)		
Interventions	Massage (yes)	34.4	(596)		
	Manual manipulation (yes)	37.9	(657)		
	Physical modalities (yes)	12.0	(208)		
	Exercise therapy (yes)	65.8	(1,141)		
	Information/advice (yes)	27.1	(469)		
Number of treatment sessions				9.9	(6.6)

^1 ^[27]

^2 ^recurrent complaint is defined as a complaint appearing after a complaint-free episode lasting at least four weeks and at most two years

^3 ^for the same or other complaint

### Data analysis

Descriptive statistics were calculated for the characteristics of the patients, the therapists, and the practices, and for the number of treatment sessions per treatment episode. Data were aggregated at the level of treatment episodes. Software-program SPSS 11.5 was used for the descriptive analysis. Missing value analyses showed four categorical variables with over 10% missing cases and a category designated as "unknown" was added for those variables. In the case of the other variables, the missing values were recoded to the mean (continuous variables) or most frequent value (categorical variables).

Data were analyzed by means of multilevel regression analysis to determine which variables were associated with the number of treatment sessions per treatment episode. Multilevel analysis was used because the data had an intrinsically hierarchical nature; the patients (level 1) are nested in the sample of physical therapists (level 2), who are nested in physical therapy practices (level 3). The data were not based on independent observations, therefore, which violates a major assumption of traditional regression analysis. Multiple levels are taken into account in multilevel analysis and variation can be split between levels.

Bivariate correlations between all predictor variables were examined to check for high correlations before starting the multilevel analysis. The therapists' age and the time since their graduation showed a correlation of 0.80 and so only the therapists' age was included in the analysis.

The multilevel analysis was carried out using MLwiN 1.1 software. The order of adding predictor variables to the model was determined by their level, as described above.

The analysis was carried out in 2 steps. An "intercept-only model" was made first. This is a model without any predictor variables, which establishes the contribution of each level to the variation in the number of treatment sessions. In the next step, all predictor variables were added. The multilevel analysis was done with three dependent variables: viz. the raw number of sessions, a log-linear transformation and a dataset in which the extreme values had been left out.

Indicator coding was used for categorical predictor variables, with the first category in each group treated as the reference group. The continuous predictor variables "patient's age" and "number of patients per therapist per month" were centered around their mean. The contribution of each predictor variable was expressed in a regression coefficient (B) and a standard error (SE). If their quotient is greater than 1.96 or smaller than -1.96, the coefficient is statistically significant (level of significance is 0.05) [20].

## Results

The three different analyses yielded similar results. Since analyses containing log-transformation will be difficult for the reader to interpret, only the results on the raw number of sessions will be shown.

### Number of treatment sessions per treatment episode

The mean number of physical therapy treatment sessions in patients referred with non-specific LBP was 9.9 (SD 6.6; median 9.0; minimum 1; maximum 67).

### Variance components in intercept-only model

As shown in Table 5, most of the variance in treatment sessions was located among patients (88.4%); 4.4% of the total variance was located among therapists and 7.2% was located among practices. The mean number of treatment sessions, adjusted for therapists and practices, was 10.0. Using the intercept and the variance component at practice level, the mean number of treatment sessions in 95% of the practices was calculated to be between 6.6 and 13.4.

**Table 5:** Distribution of variation in the number of physical therapy treatment sessions in patients with non-specific LBP among different levels (practices, therapists, and patients). Results of the intercept-only model (n = 1,733)

		(SE)	%	P
Intercept	10.03	(0.37)		
Deviance	11,299.52			
				
*Variance*				
Practice level	3.06	(1.28)	7.2%	0.016
Therapist level	1.88	(0.91)	4.4%	0.038
Patient level	37.75	(1.314)	88.4%	<0.001
Total	42.70		100.0%	

### Contribution of predictor variables in the final model

The contribution of the various predictor variables in the last step of the analyses is expressed in regression coefficients and standard errors in Table 6.

**Table 6:** Hierarchical regression analysis of predictors of the number of physical therapy treatment sessions in patients with non-specific LBP (n = 1,733)

		B	(SE)
Intercept		9.300	(1.28)
			
**Patient level**			
Age (years)		***0.04	(0.01)
Female (ref. Male)		***1.90	(0.42)
Education level:	Middle (ref. low)	0.62	(0.40)
	High (ref. low)	-0.76	(0.52)
	Unknown (ref. low)	-0.28	(0.42)
Health insurance:	Private (ref. public)	*-0.84	(0.38)
	Unknown (ref. public)	0.34	(0.42)
High urbanization rate (ref. low)		0.10	(0.53)
			
**Complaint level**			
Chronic complaints (ref. acute)		***2.27	(0.44)
Female*chronic complaints		**-1.79	(0.59)
Recurrent complaint (ref. no)		-0.34	(0.34)
Previous therapy (ref. no)		***1.17	(0.35)
Referral by medical specialist (ref. GP)		***4.18	(0.77)
			
**Treatment level**			
Treatment goal	Maintaining body position	0.09	(0.49)
	Changing body position	0.31	(0.46)
	Functions of mobility	0.08	(0.43)
	Functions of muscles	0.32	(0.51)
	Pain	1.23	(0.64)
Interventions	Massage	0.71	(0.37)
	Manual manipulation	-0.38	(0.36)
	Physical modalities	*1.13	(0.48)
	Exercise therapy	**1.03	(0.35)
	Information/advice	-0.33	(0.38)
			
**Therapist and practice level**			
Female (ref. male)		*-1.23	(0.57)
Aged > 45 years (ref. < 45 years)		***-2.01	(0.51)
Manual therapist (ref. no)		*-1.44	(0.60)
Patient-related working hours per week	20–40 (ref. < 20)	-0.48	(0.61)
	> 40 (ref. < 20)	*1.80	(0.87)
Additional training in LBP (ref. no)		**-1.47	(0.51)
Additional training in LBP guideline (ref. no)		0.39	(0.47)
Feasibility LBP guideline	> 7 (ref. < 7)	0.09	(0.48)
	Unknown (ref. < 7)	-0.31	(0.65)
Number of LBP patients per therapist per month		-0.14	(0.18)
Group practice (ref. single-handed)		0.14	(0.80)
			
*Deviance*		*11,106.46*	

* = P < 0.05; ** = P < 0.01; *** = P < 0.001

The influence of the characteristics with regard to the complaints appeared to be most powerful when all predictor variables were included for hierarchical linear regression analysis. Three out of four variables were related to the number of treatment sessions. Patients with sub-acute or chronic complaints received 2.3 sessions more compared to patients with acute complaints when all other variables were held constant; patients who were referred by a medical specialist received 4.2 sessions more compared to patients referred by a general practitioner; and patients who had prior therapy for the same or other complaints received 1.2 sessions more compared to patients who did not have prior therapy.

Demographic variables also had a statistically significant relationship to the number of treatment sessions. Older patients, female patients, and patients with public health insurance were treated more often than other patients. The level of education did not have a statistically significant relationship to the number of treatment sessions when all other predictor variables were controlled.

Treatment goals did not have a statistically significant relationship to the number of physical therapy treatment sessions. Two out of five interventions did show an association with the number of treatment sessions; patients in whom exercise therapy or physical modalities are part of the treatment are treated in one session more than other patients.

Although most of the variance was located among patients, characteristics of the therapists were also shown to be related to the number of treatment sessions. Patients treated by a manual therapist received 1.4 sessions fewer than patients treated by other physical therapists. Therapists with additional training in LBP treated their patients in 1.5 sessions less than therapists without additional training. Female therapists and older therapists treated their patients in fewer sessions than younger and male therapists. Finally, therapists working more than 40 hours a week treated their patients in more sessions than therapists working less than 20 hours a week.

### Explained variance in final model

Compared to the intercept-only model, the final model explained 13.4% of the variance (Table 7); 8.7% was explained at patient level, where most of the variance was located. The variance at practice level decreased by 22.2%, while the variance at therapist level disappeared almost entirely (decrease 93.2%). In the final model, in which all predictor variables were added to the model, 93% of the variance was located among patients (not in table); the remaining variance was mainly located among practices.

**Table 7:** Distribution of variation in the number of physical therapy treatment sessions in patients with non-specific LBP among different levels (practices, therapists, and patients). Results of step two in the analyses (n = 1,733)

	Variance	(SE)	*% of explained variance in relation to the intercept-only model*
Practice level	2.38	(0.83)	*22.2*
Therapist level	0.13	(0.43)	*93.2*
Patient level	34.48	(1.20)	*8.7*
Total	36.98		*13.4*

## Discussion

This study confirms that there is substantial variation in the number of physical therapy treatment sessions for patients with LBP and most of this variance is located among patients. A combination of various factors explains 13.4% of the variance in the number of physical therapy treatment sessions.

To our knowledge, this is the first study in which the variation in the number of physical therapy treatment sessions for LBP among patients, therapists and practices has been estimated simultaneously. The findings have major implications for the quality of care agenda in physical therapy.

Most of the variance by far is located at patient level. Demographic factors and factors relating to the complaints explained the major part of the variance, compared with factors relating to the treatment and the therapists. The positive association between the patient's age and the number of treatment sessions is in accordance with the literature [5,14], as is the effect of the patient's gender [14]. The duration of the complaint, prior therapy, and the specialization of the referrer are also related to the number of treatment sessions. Although there might be other explanations as well, this finding is in agreement with the assumption that these factors are related to the severity of the complaint. On grounds of equity, it is appropriate that the severity of the complaint is related to the number of physical therapy treatment sessions. The same is true of the relationship between the interventions and the number of treatment sessions, since it has been suggested that the contents of care are related to the severity of the complaints [2,7]. Jette et al. (1996) were able to show that outcomes were associated with the use of some types of physical therapy treatment in patients with spinal impairments [8]. As the outcome of care was not investigated in the current study, it might be interesting to carry out further investigation into the relationship between the content of the treatment, the number of treatment sessions and the outcome.

Factors at therapist level, such as their age, gender and specialty, were also associated with the number of treatment sessions, as were demographic factors and factors relating to the treatment and the complaints. It is questionable whether associations with factors at therapist level are desirable. It is suggested in the literature that practice style differences flourish in an environment of professional uncertainty [21,22]. The Dutch physical therapy guideline for LBP was published in 2001 to reduce professional uncertainty [19]. The effects of this publication on physical therapy practice might not be completely visible, since our results are based on data from patients treated between 2002 and 2004. The corresponding variable, however, does not show a relationship to the number of treatment sessions. Furthermore, the variation located at practice level might indicate a (conscious or unconscious) practice policy regarding the number of treatment sessions. This is in accordance with the assumption that individual practitioners are embedded within medical groups and that shared circumstances channel the behavior of the group members, as stated by Westert et al. (1999) [22].

In the current study, it proved possible to explain 13% of the variance. Although this percentage seems rather low, it is consistent with other studies carried out in health care professions [14,23,24]. Dunlop et al. (2000) studied the role of socio-economic status in the differential use of physician services and were able to explain between 9% and 20% of the variance in the various analyses [23]. Kersnik et al. (2001) investigated predictors of frequent attendance in general practice and explained 20% of the variance [24]. Finally, Zuijderduin et al. (1995) studied factors related to the number of treatment sessions and were able to explain 16% of the variance in the number of treatment sessions [14].

It is necessary to gain more insight into the variation in the number of treatment sessions in order to increase the transparency of physical therapy care and to increase its quality. What we particularly need to know is whether the unexplained variation is appropriate or not, as quality policy should be aimed at decreasing variance caused by inappropriate factors. Unexplained variation could consist of appropriate factors, such as psychosocial characteristics. Coping style, for example, is predictive of the ability to control or adjust pain [25] and a higher ability to control pain might result in a lower number of physical therapy treatment sessions. Furthermore, some LBP patients have high levels of fear avoidance beliefs, which result in avoidance behavior. Avoidance behavior is perceived to be a maladaptive response, as it is associated with negative psychological consequences (e.g. exaggerated pain perception) and negative physiological consequences (e.g. decreased range of spine motion) [26]. This reaction is likely to be associated with a higher number of treatment sessions. The extent to which these factors are indeed related to the number of physical therapy treatment sessions is unclear as yet, however. In addition to psychosocial factors, the ability to learn motor behavior might also influence the number of physical therapy treatment sessions. Patients with a low ability to learn motor behavior will need more treatment sessions than patients with a high ability to learn motor behavior. Furthermore, a patient with a high baseline disability will need more treatment sessions than a patient with a low baseline disability. On the other hand, inappropriate factors, such as demands made by a patient that have no clinical relevance, might also be part of the unexplained variation. It will be a challenge for future investigations to study the effects of the above-mentioned characteristics as well.

The mean number of treatment sessions is ten in the current study, but comparisons with international literature suggest that the mean number of treatment sessions varies. One study in Northern Ireland showed a median number of five treatment sessions for patients with LBP [4], while a study in the United States of America showed a mean number of eleven treatment sessions [6]. In the Dutch situation, the mean number of treatment sessions is located around the number that is eligible for reimbursement by public health insurance funds.

The limitations of the current study include its reliance on therapists to accurately record relevant data, but we expect only minimal inaccuracies in the data for two reasons. Firstly, the participating therapists charge the health care funds electronically for the treatment sessions provided. In the current study, a quantity of the data collected has been filtered out of this reimbursement data. Secondly, missing data or wrong data are corrected by means of standardized quality control. Another limitation of the study is the possibility that the participating therapists are a subgroup of Dutch therapists, i.e. therapists working in computerized practices and therapists that were willing to participate. Basic characteristics of the participants, however, like gender, age, and years since graduation, are comparable to all Dutch therapists.

## Conclusion

In summary, our results suggest that the number of physical therapy treatment sessions in patients referred with non-specific LBP mainly depends on characteristics at patient level. The greater part of the clinical variation was not explained, however, which means that additional research focusing on psychosocial factors is necessary for a progressive increase in the transparency of care.

## Competing interests

The author(s) declare that they have no competing interests.

## Authors' contributions

IS participated in the design of the study, performed the statistical analyses and drafted the manuscript. RW conceived of the study, participated in the design and helped to draft the manuscript. PG participated in the design of the study. WB, JD and EE participated in the design of the study and helped to draft the manuscript. All authors read and approved the final manuscript.

## Pre-publication history

The pre-publication history for this paper can be accessed here:



## References

[B1] Armstrong MP, McDonough SM, Baxter GD (2003). Clinical guidelines versus clinical practice in the management of low back pain. Int J Clin Pract.

[B2] Dekker J, van Baar ME, Curfs EC, Kerssens JJ (1993). Diagnosis and treatment in physical therapy: an investigation of their relationship. Phys Ther.

[B3] Foster NF, Thompson KA, Baxter GD, Allen JM (1999). Management of Nonspecific Low Back Pain by Physiotherapists in Britain and Ireland. A descriptive Questionnaire of Current Clinical Practice. Spine.

[B4] Gracey JH, McDonough SM, Baxter GD (2002). Physiotherapy Management of Low Back Pain. A Survey of Current Practice in Northern Ireland. Spine.

[B5] Hendriks HJM (2000). Physiotherapist's Consultation in General Practice. PhD thesis.

[B6] Jette AM, Smith K, Haley MS, Davis KD (1994). Physical Therapy Episodes of Care for Patients with Low Back Pain. Phys Ther.

[B7] Jette AM, Delitto A (1997). Physical therapy treatment choices for musculoskeletal impairments. Phys Ther.

[B8] Jette DU, Jette AM (1996). Physical therapy and health outcomes in patients with spinal impairments. Phys Ther.

[B9] Mielenz TJ, Carey TS, Dyrek DA, Harris BA, Garrett JM, Darter JD (1997). Physical therapy utilization by patients with acute low back pain. Phys Ther.

[B10] van der Valk R, Dekker J, van Baar ME (1995). Physical Therapy for Patients with Back Pain. Physiotherapy.

[B11] Andersen RM (1995). Revisiting the Behavioral Model and access to medical care: does it matter?. J Health Soc Behav.

[B12] Hannan TJ (1999). Variation in health care – the roles of the electronic medical record. Int J med Inform.

[B13] Mitchell JM, de Lissovoy G (1997). A comparison of resource use and cost in direct access versus physician referral episodes of physical therapy. Phys Ther.

[B14] Zuijderduin W, Dekker J, Abrahamse H (1995). Determinanten van de omvang van de behandeling in de extramurale fysiotherapie. [Determinants of the number of sessions in a physical therapeutic treatment]. Tijdschr Soc Gezondheidsz.

[B15] Hingstman L, Kenens R (2002). Cijfers over fysiotherapeuten in de eerste lijn 2001 [Data on physical therapists in primary care 2001] Utrecht: NIVEL.

[B16] Dekker J, van Ravensberg D, van den Ende E, Oostendorp R (1998). De beperkende maatregel fysiotherapie, oefentherapie Cesar en oefentherapie-Mensendieck en het Amsterdams Dienstenmodel: samenvatting van het evaluatie-onderzoek Deelrapport 4 [The restrictive measure for physical therapy, remedial therapy (Cesar and Mensendieck) and the Amsterdam 'services model': summary of the evaluation research Part 4] Utrecht: Nivel/Npi.

[B17] Hingstman L, Kenens R, van der Windt W, Talma HF, Meihuizen HE, de Voogd-Hamelink AM (2001). Rapportage arbeidsmarkt zorg en welzijn 2001 Hoofdrapport [Report on the labour market for health care and welfare Main report] Tilburg: OSA publicatie ZW 21.

[B18] Verheij R, Jabaaij L, de Bakker D, Abrahamse H, van den Hoogen H, Braspenning J, van Althuis T, Rutten R (2002). LINH jaarrapport 2001 cijfers uit het Landelijk Informatie Netwerk Huisartsenzorg: contacten, verwijzingen en voorschrijven in de huisartsenpraktijk [LINH 2001 annual report: encounters, referrals and prescribing in General Practice] Utrecht: Nivel.

[B19] Bekkering GE, Hendriks HJM, Koes BW, Oostendorp RAB, Ostelo RWJG, Thomassen JMC, van Tulder MW (2003). Dutch Physiotherapy Guidelines for Low Back Pain. Physiotherapy.

[B20] Hox JJ (1995). Applied multilevel analysis.

[B21] Long MJ (2002). An explanatory model of medical practice variation: a physician resource demand perspective. J Eval Clin Pract.

[B22] Westert GP, Groenewegen PP (1999). Medical Practice Variations: changing the theoretical approach. Scand J Public Health.

[B23] Dunlop S, Coyte PC, McIsaac W (2000). Socio-economic status and the utilization of physicians' services: results from the Canadian National Population Health Survey. Soc Sci Med.

[B24] Kersnik J, Švab I, Vegnuti M (2001). Frequent attenders in general practice: quality of life, patient satisfaction, use of medical services and GP characteristics. Scand J Prim Health Care.

[B25] Rosenstiel AK, Keefe FJ (1983). The use of coping strategies in chronic low back pain patients' relationship to patient characteristics and current adjustment. Pain.

[B26] George SZ, Bialosky JE, Fritz JM (2004). Physical therapist management of a patient with acute low back pain and elevated fear-avoidance beliefs. Phys Ther.

[B27] Statistics Netherlands. http://www.cbs.nl..

